# Creativity, Critical Thinking, Communication, and Collaboration: Assessment, Certification, and Promotion of 21st Century Skills for the Future of Work and Education

**DOI:** 10.3390/jintelligence11030054

**Published:** 2023-03-15

**Authors:** Branden Thornhill-Miller, Anaëlle Camarda, Maxence Mercier, Jean-Marie Burkhardt, Tiffany Morisseau, Samira Bourgeois-Bougrine, Florent Vinchon, Stephanie El Hayek, Myriam Augereau-Landais, Florence Mourey, Cyrille Feybesse, Daniel Sundquist, Todd Lubart

**Affiliations:** 1 Faculty of Philosophy, University of Oxford, Oxford OX2 6GG, UK; 2 International Institute for Competency Development, 75001 Paris, France; 3 LaPEA, Université Paris Cité and Univ Gustave Eiffel, 92100 Boulogne-Billancourt, France; 4 Institut Supérieur Maria Montessori, 94130 Nogent-Sur-Marne, France; 5 LaPEA, Univ Gustave Eiffel and Université Paris Cité, CEDEX, 78008 Versailles, France; 6 Strane Innovation, 91190 Gif-sur-Yvette, France; 7 AFNOR International, 93210 Saint-Denis, France; 8 Centre Hospitalier Guillaume Regnier, Université de Rennes 1, 35200 Rennes, France

**Keywords:** 21st century skills, 4Cs, assessment, certification, collaboration, communication, creativity, critical thinking, education, future of work, games, labelization, soft skills, training

## Abstract

This article addresses educational challenges posed by the future of work, examining “21st century skills”, their conception, assessment, and valorization. It focuses in particular on key soft skill competencies known as the “4Cs”: creativity, critical thinking, collaboration, and communication. In a section on each C, we provide an overview of assessment at the level of individual performance, before focusing on the less common assessment of *systemic support* for the development of the 4Cs that can be measured at the institutional level (i.e., in schools, universities, professional training programs, etc.). We then present the process of official assessment and certification known as “labelization”, suggesting it as a solution both for establishing a publicly trusted assessment of the 4Cs and for promoting their cultural valorization. Next, two variations of the “International Institute for Competency Development’s 21st Century Skills Framework” are presented. The first of these comprehensive systems allows for the assessment and labelization of the extent to which development of the 4Cs is supported by a formal educational program or institution. The second assesses informal educational or training experiences, such as playing a game. We discuss the overlap between the 4Cs and the challenges of teaching and institutionalizing them, both of which may be assisted by adopting a dynamic interactionist model of the 4Cs—playfully entitled “Crea-Critical-Collab-ication”—for pedagogical and policy-promotion purposes. We conclude by briefly discussing opportunities presented by future research and new technologies such as artificial intelligence and virtual reality.

## 1. Introduction

There are many ways of describing the massive educational challenges faced in the 21st century. With the appearance of computers and digital technologies, new means of interacting between people, and a growing competitiveness on the international level, organizations are now requiring new skills from their employees, leaving educational systems struggling to provide appropriate ongoing training. Indeed, according to the World Economic Forum’s 2020 “Future of Jobs Report”, studying 15 industries in 26 advanced and emerging countries, up to 50% of employees will need some degree of “reskilling” by 2025 (World Economic Forum 2020). Although many national and international educational efforts and institutions now explicitly put the cultivation of new kinds of skills on their educational agendas, practical means of assessing such skills remains underdeveloped, thus hampering the valorization of these skills and the development of guidance for relevant pedagogy (Care et al. 2018; Vincent-Lancrin et al. 2019; for overviews and discussion of higher education in global developmental context, see Blessinger and Anchan 2015; Salmi 2017).

This article addresses some of these challenges and related issues for the future of education and work, by focusing on so-called “21st Century Skills” and key “soft skills” known as the “4Cs” (creativity, critical thinking, communication, and collaboration), more particularly. It begins with a brief discussion of these skills, outlining their conceptual locations and potential roles in the modern educational context. A section on each “C” then follows, defining the C, summarizing research and methods for its scientific assessment at the individual level, and then outlining some means and avenues at the systemic level for fostering its development (e.g., important aspects of curriculum, institutional structure, or of the general environment, as well as pedagogical methods) that might be leveraged by an institution or program in order to promote the development of that C among its students/trainees. In the next section, the certification-like process of “labelization” is outlined and proposed as one of the best available solutions both for valorizing the 4Cs and moving them towards the center of the modern educational enterprise, as well as for benchmarking and monitoring institutions’ progress in fostering their development. The International Institute for Competency Development’s 4Cs Framework is then outlined as an example of such a comprehensive system for assessing and labelizing the extent to which educational institutions and programs support the development of the 4Cs. We further demonstrate the possibility of labelizing and promoting support for the development of the 4Cs by activities or within less formal educational settings, presenting a second framework for assessment of the 4Cs in games and similar training activities. Our discussion section begins with the challenges to implementing educational change in the direction of 21st century skills, focusing on the complex and overlapping nature of the 4Cs. Here, we propose that promoting a “Dynamic Interactionist Model of the 4Cs” not only justifies grouping them together, but it might also assist more directly with some of the challenges of pedagogy, assessment, policy promotion, and ultimately, institutionalization, faced by the 4Cs and related efforts to modernize education. We conclude by suggesting some important future work for the 4Cs individually and also as an interrelated collective of vital skills for the future of education and work.

### “21st Century Skills”, “Soft Skills”, and the “4Cs”

**Figure 1:**
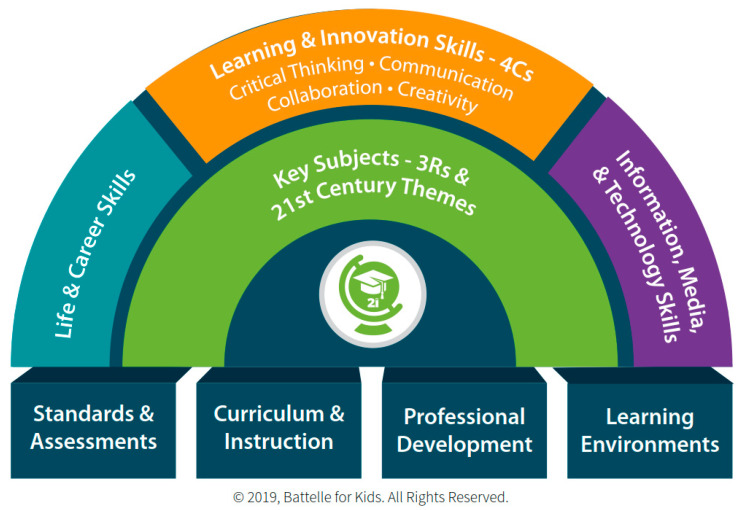
The P21 Framework for 21st Century Learning. (© 2019, Battelle for Kids. All Rights Reserved. https://www.battelleforkids.org/; accessed on 17 January 2023).

For 40 years, so-called “21st century skills” have been promoted as those necessary for success in a modern work environment that the US Army War College (Barber 1992) has accurately described as increasingly “VUCA”—“volatile, uncertain, complex and ambiguous”. Various lists of skills and competencies have been formulated on their own or as part of comprehensive overarching educational frameworks. Although a detailed overview of this background material is outside the scope of this article (see Lamri et al. 2022; Lucas 2022 for summaries), one of the first prominent examples of this trend was the Partnership for 21st Century Skills (P21), whose comprehensive “Framework for 21st Century Learning” is presented in Figure 1 (Battelle for Kids 2022). This framework for future-oriented education originated the idea of the “4Cs”, placing them at its center and apex as “Learning and Innovation Skills” that are in need of much broader institutional support at the foundational level in the form of new standards and assessments, curriculum and instructional development, ongoing professional development, and appropriately improved learning environments (Partnership for 21st Century Skills 2008). These points are also consistent with the approach and assessment frameworks presented later in this article.

Other important organizations such as the World Economic Forum (2015) have produced similar overarching models of “21st century skills’’ with the 4Cs at their center, but the term “21st century skills’’ has been rightly criticized for a several reasons: the skills referred to are not actually all unique to, or uniquely important to, the 21st century, and it is a term that is often used more as an advertising or promotional label for systems that sometimes conflate and confuse different kinds of skills with other concepts that users lump together (Lucas 2019). Indeed, though there is no absolute consensus on the definition of a “skill”, they are often described as being multidimensional and involve the ability to solve problems in context and to perform tasks using appropriate resources at the right time and in the right combination (Lamri and Lubart 2021). At its simplest, a skill is a “learned capacity to do something useful” (Lucas and Claxton 2009), or an ability to perform a given task at a specified performance level, which develops through practice, experience. and training (Lamri et al. 2022).

The idea of what skills “are’’, however, has also evolved to some extent over time in parallel to the nature of the abilities required to make valued contributions to society. The digital and information age, in particular, has seen the replacement by machines of much traditional work sometimes referred to as “hard skills’’—skills such as numerical calculation or driving, budget-formulating, or copyediting abilities, which entail mastery of fixed sets of knowledge and know-how of standard procedures, and which are often learned on the job. Such skills are more routine, machine-related, or technically oriented and not as likely to be centered on human interaction. In contrast, the work that has been increasingly valued in the 21st century involves the more complex, human interactive, and/or non-routine skills that Whitmore (1972) first referred to as “soft skills”.

Unfortunately, researchers, educators, and consultants have defined, redefined, regrouped, and expanded soft skills—sometimes labeling them “transversal competencies”, “generic competencies”, or even “life skills” in addition to “21st century skills”—in so many different ways within and across different domains of research and education (as well as languages and national educational systems) that much progress towards these goals has literally been “lost in translation” (Cinque 2016).

Indeed, there is also a long-standing ambiguity and confusion between the terms “competency” (also competence) and “skill” due to their use across different domains (e.g., learning research, education, vocational training, personnel selection) as well as different epistemological backgrounds and cultural specificities (Drisko 2014; Winterton et al. 2006; van Klink and Boon 2003). The term “competency” is, however, often used as a broader concept that encompasses skills, abilities, and attitudes, whereas, in a narrower sense, the term “skill” has been defined as “goal-directed, well-organized behavior that is acquired through practice and performed with economy of effort” (Proctor and Dutta 1995, p. 18). For example, whereas the command of a spoken language or the ability to write are skills (hard skills, to be precise), the ability to communicate effectively is a competence that may draw on an individual’s knowledge of language, writing skills, practical IT skills, and emotional intelligence, as well as attitudes towards those with whom one is communicating (Rychen and Hersch 2003). Providing high-quality customer service is a competency that relies on listening skills, social perception skills, and contextual knowledge of products. Beyond these potential distinctions, the term “competency” is predominant in Europe, whereas “skill” is more commonly used in the US. Yet it also frequently occurs that both are used as rough synonyms. For example, Voogt and Roblin (2012, p. 299) examine the “21st century competences and the recommended strategies for the implementation of these skills”, and Graesser et al. (2022, p. 568) state that twenty-first-century skills “include self-regulated learning, collaborative problem solving, communication (…) and other competencies”. In conclusion, the term “competencies” is often used interchangeably with “skills” (and can have a particularly large overlap with “soft skills”), but it is also often considered in a broader sense as a set of skills, knowledge, and attitudes that, together, meet a complex demand (Ananiadoui and Claro 2009). From this perspective, one could argue that the 4Cs, as complex, “higher-order” soft skills, might best be labeled competencies. For ease and convenience, however, in this text, we consider the two terms interchangeable but favor the term “skills”, only using “competency” in some instances to avoid cumbersome repetition.

Even having defined soft skills as a potentially more narrow and manageable focus, we are still aware of no large-scale study that has employed a comprehensive enough range of actual psychometric measures of soft skills in a manner that might help produce a definitive empirical taxonomy. Some more recent taxonomic efforts have, however, attempted to provide additional empirical grounding for the accurate identification of key soft skills (see e.g., Joie-La Marle et al. 2022). Further, recent research by JobTeaser (see Lamri et al. 2022) surveying a large, diverse sample of young workers about a comprehensive, systematic list of soft skills *as actually used in their professional roles* represents a good step towards some clarification and mapping of this domain on an empirical basis. Despite the fact that both these studies necessarily involved assumptions and interpretive grouping of variables, the presence and importance of the 4Cs as higher-order skills is evident in both sets of empirical results.

Various comprehensive “21st century skills” systems proposed in the past without much empirical verification also seem to have been found too complex and cumbersome for implementation. The 4Cs, on the other hand, seem to provide a relatively simple, persuasive, targetable core that has been found to constitute a pedagogically and policy-friendly model by major organizations, and that also now seems to be gaining some additional empirical validity. Gathering support from researchers and industry alike, we suggest that the 4Cs can be seen as highest-level transversal skills—or “meta-competencies”—that allow individuals to remain competent and to develop their potential in a rapidly changing professional world. Thus, in the end, they may also be one of the most useful ways of summarizing and addressing the critical challenges faced by the future of work and education (National Education Association 2011).

Taking them as our focus, we note, however, that the teaching and development of the 4Cs will require a complex intervention and mobilization of educational and socio-economic resources—both a major shift in pedagogical techniques and even more fundamental changes in institutional structures (Ananiadoui and Claro 2009). One very important issue for understanding the 4Cs and their educational implementation related to this, which can simultaneously facilitate their teaching but be a challenge for their assessment, is the multidimensionality, interrelatedness, and transdisciplinary relevance of the 4Cs. Thus, we address the relationships between the Cs in the different C sections and later in our Discussion, we present a “Dynamic Interactionist Model of the 4Cs’’ that we hope will assist in their understanding, in the further development of pedagogical processes related to them, and in their public promotion and related policy. Ultimately, it is partly due to their complexity and interrelationships, we argue, that it is important and expedient that the 4Cs are taught, assessed, and promoted together.

## 2. The 4Cs, Assessment, and Support for Development

### 2.1. Creativity

In psychology, creativity is usually defined as the capacity to produce novel, original work that fits with task constraints and has value in its context (for a recent overview, see Lubart and Thornhill-Miller 2019). This basic definition, though useful for testing and measurement, is largely incomplete, as it does not contain any information about the individual or groups doing the creating or the nature of physical and social contexts (Glăveanu 2014). Moreover, Corazza (2016) challenged this standard definition of creativity, arguing that as it focuses solely on the existence of an original and effective outcome, it misses the dynamics of the creative process, which is frequently associated with periods of creative inconclusiveness and limited occasions of creative achievements. To move away from the limitations of the standard definition of creativity, we can consider Bruner’s description of creativity as “figuring out how to use what you already know in order to go beyond what you currently think” (p. 183 in Weick 1993). This description echoes the notion of potential, which refers to a latent state that may be put to use if a person has the opportunity.

Creativity is a multifaceted phenomenon that can be approached from many different angles. There are three main frameworks for creativity studies: the 4Ps (Rhodes 1961), the 5As (Glăveanu 2013), and the 7Cs model (Lubart 2017). These frameworks share at least four fundamental and measurable dimensions: the act of creating (process), the outcome of the creative process (product), the characteristics of creative actor(s) enacting the process (person), and the social and physical environment that enable or hinder the creative process (press). Contrary to many traditional beliefs, however, creativity can be trained and taught in a variety of different ways, both through direct, active teaching of creativity concepts and techniques and through more passive and indirect means such as the development of creativity-supporting contexts (Chiu 2015; Thornhill-Miller and Dupont 2016). Alongside intelligence, with which it shares some common mechanisms, creativity is now recognized as an indispensable element for the flexibility and adaptation of individuals in challenging situations (Sternberg 1986).

#### 2.1.1. Individual Assessment of Creativity

Drawing upon previous efforts to structure creativity research, Batey (2012) proposed a taxonomic framework for creativity measurement that takes the form of a three-dimensional matrix: (a) the level at which creativity may be measured (the individual, the team, the organization, and the culture), (b) the facets of creativity that may be assessed (person/trait, process, press, and product), and (c) the measurement approach (objective, self-rating, other ratings). It is beyond the scope of this article to offer a literature review of all these dimensions, but for the purposes of this paper, we address some important aspects of individual-level and institutional-level assessment here.

Assessing creativity at an individual level encompasses two major approaches: (1) creative accomplishment based on production and (2) creative potential. Regarding the first approach focusing on *creative accomplishment*, there are at least four main assessment techniques (or tools representing variations of assessment techniques): (a) the historiometric approach, which applies quantitative analysis to historically available data (such as the number of prizes won or times cited) in an effort to understand eminent, field-changing creativity (Simonton 1999); (b) the Consensual Assessment Technique (CAT) (Amabile 1982), which offers a method for combining and validating judges’ subjective evaluations of a set of (potentially) creative productions or ideas; (c) the Creative Achievement Questionnaire (Carson et al. 2005), which asks individuals to supply a self-reported assessment of their publicly recognizable achievement in ten different creative domains; and (d) the Inventory of Creative Activities and Achievements (ICAA) (Jauk et al. 2014; Diedrich et al. 2018), which includes self-report scales assessing the frequency of engagement in creative activity and also levels of achievement in eight different domains.

The second major approach to individual assessment is based on *creative potential,* which measures the cognitive abilities and/or personality traits that are important for creative work. The two most popular assessments of creative potential are the Remote Associations Test (RAT) and the Alternative Uses Task (AUT). The RAT, which involves identifying the fourth word that is somehow associated with each of three given words, underscores the role that the ability to convergently associate disparate ideas plays as a key capacity for creativity. In contrast, the AUT, which requires individuals to generate a maximum number of ideas based on a prompt (e.g., different uses for a paperclip), is used to assess divergent thinking capacity. According to multivariate models of creative potential (Lubart et al. 2013), there are cognitive factors (e.g., divergent thinking, mental flexibility, convergent thinking, associative thinking, selective combination), conative factors (openness, tolerance of ambiguity, intuitive thinking, risk taking, motivation to create), and environmental factors that all support creativity. Higher creative potential is predicted by having more of the ingredients for creativity. However, multiple different profiles among a similar set of these important ingredients exist, and their weighting for optimal creative potential varies according to the profession, the domain, and the task under consideration. For example, Lubart and Thornhill-Miller (2021) and Lubin et al. (forthcoming) have taken this creativity profiling approach, exploring the identification and training of the components of creative potential among lawyers and clinical psychologists, respectively. For a current example of this sort of comprehensive, differentiated measurement of creative potential in adults in different domains and professions, see CreativityProfiling.org. For a recent battery of tests that are relevant for children, including domain-relevant divergent-exploratory and convergent-integrative tasks, see Lubart et al. (2019). Underscoring the growing recognition of the importance of creativity assessment, measures of creative potential for students were introduced internationally for the first time in the PISA 2022 assessment (OECD 2019a).

#### 2.1.2. Institutional and Environmental Support for Development of Creativity

The structural support that institutions and programs can provide to promote the development of creativity can be described as coming through three main paths: (1) through design of the physical environment in a manner that supports creativity, (2) through teaching about creativity, the creative process, and creativity techniques, and (3) through training opportunities to help students/employees develop personal habits, characteristics, and other ingredients associated with creative achievement and potential.

Given the multi-dimensionality of the notion of creativity, the environment can positively influence and help develop creative capacities. Studies have shown that the physical environment in which individuals work can enhance their positive emotions and mood and thus their creativity. For example, stimulating working environments might have unusual furniture and spaces that have natural light, windows open to nature, plants and flowers, a relaxing atmosphere and colors in the room (e.g., green and blue), or positive sounds (e.g., calm music or silence), as well as inspiring and energizing colors (e.g., yellow, pink, orange). Furthermore, the arrangement of physical space to promote interpersonal exchange rather than isolation, as well as the presence of tools, such as whiteboards, that support and show the value of exchange, are also important (for reviews, see Dul and Ceylan 2011; Samani et al. 2014).

Although it has been claimed that “creativity is intelligence having fun” (Scialabba 1984; Reiman 1992), for most people, opportunities for fun and creativity, especially in their work environment, appear rather limited. In fact, the social and physical environment often hinders creativity. Corazza et al. (2021)’s theoretical framework concerning the “Space-Time Continuum”, related to support for creativity, suggests that traditional education systems are an example of an environment that is “tight” both in the conceptual “space” it affords for creativity and in the available time allowed for creativity to happen—essentially leaving little room for original ideas to emerge. Indeed, though world-wide data suggest that neither money nor mere time spent in class correlate well with educational outcomes, both policies and pedagogy that direct the ways in which time is spent make a significant difference (Schleicher 2022). Research and common sense suggest that teachers, students, and employees need more space and time to invest energy in the creative process and the development of creative potential.

Underscoring the importance of teaching the creative process and creativity techniques is the demonstration, in a number of contexts, that groups of individuals who generate ideas without a specific method are often negatively influenced by their social environment. For example, unless guarded against, the presence of others tends to reduce the number of ideas generated and to induce a fixation on a limited number of ideas conforming to those produced by others (Camarda et al. 2021; Goldenberg and Wiley 2011; Kohn and Smith 2011; Paulus and Dzindolet 1993; Putman and Paulus 2009; Rietzschel et al. 2006). To overcome these cognitive and social biases, different variants of brainstorming techniques have shown positive effects (for reviews of methods, see Al-Samarraie and Hurmuzan 2018; Paulus and Brown 2007). These include: using (Osborn 1953) initial brainstorming rules (which aim to reduce spontaneous self-judgment of ideas and fear of this judgment by others); drawing attention to ideas generated by others by writing them down independently (e.g., the technique known as “brainwriting”); and requiring incubation periods between work sessions by forcing members of a problem-solving group to take breaks (Paulus and Yang 2000; Paulus and Kenworthy 2019).

It is also possible to use design methods that are structured to guide the creative process and the exploration of ideas, as well as to avoid settling on uncreative solution paths (Chulvi et al. 2012; Edelman et al. 2022; Kowaltowski et al. 2010; see Cotter et al. 2022 for a valuable survey of best practices for avoiding the suppression of creativity and fostering creative interaction and metacognition in the classroom). Indeed, many helpful design thinking-related programs now exist around the world and have been shown to have a substantial impact on creative outcomes (Bourgeois-Bougrine 2022).

Research and experts suggest the utility of many additional creativity enhancement techniques (see, e.g., Thornhill-Miller and Dupont 2016), and the largest and most rapid effects are often attributed to these more method- or technique-oriented approaches (Scott et al. 2004). More long-term institutional and environmental support for the development of creativity, however, should also include targeted training and understanding of personality and emotional traits associated with the “creative person” (e.g., empathy and exploratory habits that can expand knowledge, as well as increase tolerance of ambiguity, openness, and mental flexibility; see Lubart and Thornhill-Miller 2021). Complementing these approaches and focusing on a more systemic level, recent work conducted by the OECD exemplifies efforts aimed to foster creativity (and critical thinking) by focusing simultaneously on curriculum, educational activities, and teacher support and development at the primary, secondary, and higher education levels (see Vincent-Lancrin et al. 2019; Saroyan 2022).

### 2.2. Critical Thinking

Researchers, teachers, employers, and public policymakers around the world have long ranked the development of critical thinking (CT) abilities as one of the highest educational priorities and public needs in modern democratic societies (Ahern et al. 2019; Dumitru et al. 2018; Pasquinelli et al. 2021). CT is central to better outcomes in daily life and general problem solving (Hitchcock 2020), to intelligence and adaptability (Halpern and Dunn 2021), and to academic achievement (Ren et al. 2020). One needs to be aware of distorted or erroneous information in the media, of the difference between personal opinions and proven facts, and how to handle increasingly large bodies of information required to understand and evaluate information in the modern age.

Although much research has addressed both potentially related constructs, such as intelligence and wisdom, and lists of potential component aspects of human thought, such as inductive or deductive reasoning (for reviews of all of these, see Sternberg and Funke 2019), reaching a consensus on a definition has been difficult, because CT relies on the coordination of many different skills (Bellaera et al. 2021; Dumitru et al. 2018) and is involved in, and sometimes described from the perspective of, many different domains (Lewis and Smith 1993). Furthermore, as a transversal competency, having the skills to perform aspects of critical thinking in a given domain does not necessarily entail also having the metacognitive ability to know when to engage in which of its aspects, or having the disposition, attitude, or “mindset” that motivates one to actually engage in them—all of which are actually required to be a good critical thinker (Facione 2011).

As pointed out by the American Philosophical Association’s consensus definition, the ideal “critical thinker” is someone who is inquisitive, open-minded, flexible, fair-minded, and keeps well-informed, thus understanding different points of view and perspectives (Facione 1990b). These characteristics, one might note, are also characteristic of the “creative individual” (Facione 1990b; Lai 2011), as is the ability to imagine alternatives, which is often cited as a component of critical thinking ability (Facione 1990b; Halpern 1998). Conversely, creative production in any domain needs to be balanced by critical appraisal and thought at each step of the creative process (Bailin 1988). Indeed, it can be argued that creativity and critical thinking are inextricably linked and are often two sides of the same coin. Representing different aspects of “good thought” that are linked and develop in parallel, it seems reasonable that they should, in practice, be taught and considered together in teaching and learning (Paul and Elder 2006).

Given its complexity, many definitions of critical thinking have been offered. However, some more recent work has helpfully defined critical thinking as “the capacity of assessing the epistemic quality of available information and—as a consequence of this assessment—of calibrating one’s confidence in order to act upon such information” (Pasquinelli et al. 2021). This definition, unlike others proposed in the field (for a review, see: Bellaera et al. 2021; Liu et al. 2014), is specific (i.e., it limits the use of poorly defined concepts), as well as consensual and operational (i.e., it has clear and direct implications for the education and assessment of critical thinking skills; Pasquinelli et al. 2021; Pasquinelli and Bronner 2021). Thus, this approach assumes that individuals possess better or worse cognitive processes and strategies that make it possible to judge the reliability of the information received, by determining, for example, what the arguments provided actually are. Are the arguments convincing? Is the source of information identifiable and reliable? Does the information conflict with other information held by the individual?

It should also be noted that being able to apply critical thinking is necessary to detect and overcome the cognitive biases that can constrain one’s reasoning. Indeed, when solving a problem, it is widely recognized that people tend to automate the application of strategies that are usually relevant in similar and analogous situations that have already been encountered. However, these heuristics (i.e., automatisms) can be a source of errors, in particular, in tricky reasoning situations, as demonstrated in the field of reasoning, arithmetic problems (Kahneman 2003) or even divergent thinking tasks (Cassotti et al. 2016; for a review of biases, see Friedman 2017). Though some cognitive biases can even be seen as normal ways of thinking and feeling, sometimes shaping human beliefs and ideologies in ways that make it completely normal—and even definitely human—*not* to be objective (see Thornhill-Miller and Millican 2015), the mobilization of cognitive resources such as those involved in critical reasoning on logical bases usually makes it possible to overcome cognitive biases and adjust one’s reasoning (West et al. 2008).

According to Pasquinelli et al. (2021), young children already possess cognitive functions underlying critical thinking, such as the ability to determine that information is false. However, until late adolescence, studies have demonstrated an underdevelopment of executive functions involved in resistance to biased reasoning (Casey et al. 2008) as well as some other higher-order skills that underlie the overall critical thinking process (Bloom 1956). According to Facione and the landmark American Philosophical Association’s task force on critical thinking (Facione 1990b; Facione 2011), these components of critical thinking can be organized into six measurable skills: the ability to (1) interpret information (i.e., meaning and context); (2) analyze information (i.e., make sense of why this information has been provided, identify pro and con arguments, and decide whether we can accept the conclusion of the information); (3) make inferences (i.e., determine the implications of the evidence, its reliability, the undesirable consequences); (4) evaluate the strength of the information (i.e., its credibility, determine the trust in the person who provides it); (5) provide explanations (i.e., summarize the findings, determine how the information can be interpreted, and offer verification of the reasoning); (6) self-regulate (i.e., evaluate the strength of the methods applied, determine the conflict between different conclusions, clarify the conclusions, and verify missing elements).

#### 2.2.1. Individual Assessment of Critical Thinking

The individual assessment of critical thinking skills presents a number of challenges, because it is a multi-task ability and involves specific knowledge in the different areas in which it is applied (Liu et al. 2014; Willingham 2008). However, the literature provides several tools with which to measure different facets of cognitive functions and skills involved in the overarching critical thinking process (Lai 2011; Liu et al. 2014). Most assessments involve multiple-choice questions requiring reasoning within a particular situation based upon a constrained set of information provided. For example, in one of the most widely used tests, the California Critical Thinking Skills Test (Facione 1990a), participants are provided with everyday scenarios and have to answer multiple questions targeting the six higher-order skills described previously. Similarly, the Watson–Glaser Critical Thinking Appraisal (Watson 1980; Watson and Glaser 2010) presents test takers with passages and scenarios measuring their competencies at recognizing assumptions, evaluating arguments, and drawing conclusions. Although the Watson–Glaser is one of the oldest and most frequently used assessments internationally for hiring and promotion in professional contexts, its construct validity, like many other measures of this challenging topic, has some limitations (Possin 2014).

Less frequently, case study or experiential methods of assessment are also used. This approach may involve asking participants to reflect on past experiences, analyze the situations they faced and the way they behaved or made judgments and decisions and then took action (Bandyopadhyay and Szostek 2019; Brookfield 1997). These methods, often employed by teachers or employers on students and employees, usually involve the analysis of qualitative data that can cast doubt on the reliability of the results. Consequently, various researchers have suggested ways to improve analytic methods, and they emphasize the need to create more advanced evaluation methods (Brookfield 1997; Liu et al. 2014).

For example, Liu et al. (2014) reviewed current assessment methods and suggest that future work improves the operational definition of critical thinking, aiming to assess it both in different specific contexts and in different formats. Specifically, assessments could be contextualized within the major areas addressed by education programs (e.g., social sciences, humanities, and/or natural sciences), and the tasks themselves should be as practically connected to the “real world” as possible (e.g., categorizing a set of features, opinions, or facts based on whether or not they support an initial statement). Moreover, as Brookfield (1997) argues, because critical thinking is a social process that takes place in specific contexts of knowledge and culture, it should be assessed as a social process, therefore, involving a multiplicity of experiences, perceptions, and contributions. Thus, Brookfield makes three recommendations for improving the assessment of critical thinking that are still relevant today: (1) to assess critical thinking in specific situations, so one can study the process and the discourse related to it; (2) to involve students/peers in the evaluation of critical thinking abilities, so that the evaluation is not provided only by the instructor; and (3) to allow learners or participants in an experiment to document, demonstrate, and justify their engagement in critical thinking, because this learning perspective can provide insight into basic dimensions of the critical thinking process.

Finally, another more recent and less widely used form of assessment targets the specific executive functions that underlie logical reasoning and resistance to cognitive biases, as well as the ability of individuals to resist these biases. This form of assessment is usually done through specific experimental laboratory tasks that vary depending on the particular executive function and according to the domain of interest (Houdé and Borst 2014; Kahneman 2011; West et al. 2008).

#### 2.2.2. Institutional and Environmental Support for Development of Critical Thinking Skills

The executive functions underlying general critical thinking, the ability to overcome bias (Houdé 2000; Houdé and Borst 2014), and meta-cognitive processes (i.e., meta information about our cognitive strategies) can all be trained and enhanced by educational programs (Abrami et al. 2015; Ahern et al. 2019; Alsaleh 2020; Bellaera et al. 2021; Uribe-Enciso et al. 2017; Popil 2011; Pasquinelli and Bronner 2021; Yue et al. 2017).

Educational programs and institutions can support the development of critical thinking in several different ways. The process of developing critical thinking focuses on the interaction between personal dispositions (attitudes and habits), skills (evaluation, reasoning, self-regulation), and finally, knowledge (general and specific knowledge, as well as experience) (Thomas and Lok 2015). It is specifically in regard to skills and knowledge that institutions are well suited to develop critical thinking through pedagogical elements such as rhetoric training, relevance of information evaluation (e.g., media literacy, where and how to check information on the internet, dealing with “fake news”, etc.), deductive thinking skills, and inductive reasoning (Moore and Parker 2016). A few tools, such as case studies or concept mapping, can also be used in conjunction with a problem-based learning method, both in individual and team contexts and in person or online (Abrami et al. 2015; Carmichael and Farrell 2012; Popil 2011; Thorndahl and Stentoft 2020). According to Marin and Halpern (2011), training critical thinking should include explicit instruction involving at least the four following components and objectives: (1) working on attitudes and encouraging individuals to think; (2) teaching and practicing critical thinking skills; (3) training for transfer between contexts, identifying concrete situations in which to adopt the strategies learned; and (4) suggesting metacognition through reflection on one’s thought processes. Supporting these propositions, Pasquinelli and Bronner (2021), in a French national educational report, proposed practical advice for creating workshops to stimulate critical thinking in school classrooms, which appear relevant even in non-school intervention situations. For example, the authors suggest combining concrete examples and exercises with general and abstract explanations, rules and strategies, which can be transferred to other areas beyond the one studied. They also suggest inviting learners to create examples of situations (e.g., case studies) in order to increase the opportunities to practice and for the learner to actively participate. Finally, they suggest making the process of reflection explicit by asking the learner to pay attention to the strategies adopted by others in order to stimulate the development of metacognition.

### 2.3. Communication

In its most basic definition, communication consists of exchanging information to change the epistemic context of others. In cooperative contexts, it aims at the smooth and efficient exchange of information contributing to the achievement of a desired outcome or goal (Schultz 2010). But human communication involves multiple dimensions. Both verbal and non-verbal communication can involve large quantities of information that have to be both formulated and deciphered with a range of purposes and intentions in mind (Jones and LeBaron 2002). These dimensions of communication have as much to do with the ability to express oneself, both orally and in writing and the mastering of a language (linguistic competences), as with the ability to use this communication system appropriately (pragmatic skills; see Grassmann 2014; Matthews 2014), and with social skills, based on the knowledge of how to behave in society and on the ability to connect with others, to understand the intentions and perspectives of others (Tomasello 2005).

Like the other 4Cs, according to most authorities, communication skills are ranked by both students and teachers as skills of the highest priority for acquisition in order to be ready for the workforce in 2030 (OECD 2019b; Hanover Research 2012). Teaching students how to communicate efficiently and effectively in all the new modalities of information exchange is an important challenge faced by all pedagogical organizations today (Morreale et al. 2017). All dimensions of communication (linguistic, pragmatic, and social) are part of what is taught in school curricula at different levels. But pragmatic and social competencies are rarely explicitly taught as such. Work on social/emotional intelligence (and on its role in students’ personal and professional success) shows that these skills are both disparate and difficult to assess (Humphrey et al. 2007). Research on this issue is, however, becoming increasingly rigorous, with the potential to provide usable data for the development of science-based practice (Keefer et al. 2018). Teachers and pedagogical teams also have an important, changing role to play: they also need to master new information and communication technologies and the transmission of information through them (Zlatić et al. 2014).

Communication has an obvious link with the three other Cs. Starting with critical thinking, sound communication implies fostering the conditions for a communicative exchange directed towards a common goal, which is, at least in educational and professional contexts, based on a fair evaluation of reality (Pornpitakpan 2004). Collaboration too has a strong link with communication, because successful collaboration is highly dependent on the quality of knowledge sharing and trust that emerges between group members. Finally, creativity involves the communication of an idea to an audience and can involve high-quality communication when creative work occurs in a team context.

#### 2.3.1. Individual Assessment of Communication

Given the vast field of communication, an exhaustive list of its evaluation methods is difficult to establish. A number of methods have been reported in the literature to assess an individual’s ability to communicate non-verbally and verbally. But although these two aspects are intrinsically linked, they are rarely measured together with a single tool. Moreover, as Spitzberg (2003) pointed out, communication skills are supported by different abilities, classically conceptualized as motivational functions (e.g., confidence and goal-orientation), knowledge (e.g., content and procedural knowledge), or cognitive and socio-cognitive functions (e.g., theory of mind, verbal cognition, emotional intelligence, and empathy; McDonald et al. 2014; Rothermich 2020), implying different specific types of evaluations. Finally, producing vs. receiving communication involve different skills and abilities, which can also vary according to the context (Landa 2005).

To overcome these challenges, Spitzberg (2003) recommends the use of different assessment criteria. These criteria include the clarity of interaction, the understanding of what was involved in the interaction, the satisfaction of having interacted (expected to be higher when communication is effective), the efficiency of the interaction (the more competent someone is, the less effort, complexity, and resources will be needed to achieve their goal), its effectiveness or appropriateness (i.e., its relevance according to the context), as well as criteria relative to the quality of the dialogue (which involves coordination, cooperation, coherence, reciprocity, and mutuality in the exchange with others). Different forms of evaluation are also called for, such as self-reported questionnaires, hetero-reported questionnaires filled out by parents, teachers, or other observers, and tasks involving exposure to role-playing games, scenarios or videos (for a review of these assessment tools, see Cömert et al. 2016; Landa 2005; Sigafoos et al. 2008; Spitzberg 2003; van der Vleuten et al. 2019). Results from these tools must then be associated with others assessing underlying abilities, such as theory of mind and metacognition.

#### 2.3.2. Institutional and Environmental Support for Development of Communication Skills

Although communication appears to be a key employability skill, the proficiency acquired during studies rarely meets the expectations of employers (Jackson 2014). Communication must therefore become a priority in the training of students, beyond the sectors in which it is already known as essential (e.g., in medicine, nursing, engineering, etc.; Bourke et al. 2021; D’Alimonte et al. 2019; Peddle et al. 2018; Riemer 2007), and also through professional development (Jackson 2014). Training programs involving, for example, communication theory classes (Kruijver et al. 2000) and self-assessment tools that can be used in specific situations (Curtis et al. 2013; Rider and Keefer 2006) have had convincingly positive results. The literature suggests that interactive approaches in small groups, in which competencies are practiced explicitly in an open and feedback-safe environment, are more effective (Bourke et al. 2021; D’Alimonte et al. 2019; AbuSeileek 2012; Fryer-Edwards et al. 2006). These can take different forms: project-based work, video reviews, simulation or role-play games (see Hathaway et al. 2022 for a review; Schlegel et al. 2012). Finally, computer-assisted learning methods can be relevant for establishing a secure framework (especially, for example, when learning another language): anonymity indeed helps to overcome anxiety or social blockages linked to fear of public speaking or showing one’s difficulties (AbuSeileek 2012). Each of these methods tackles one or more dimensions of communication that must then be assessed as such, by means of tools specifically developed and adapted to the contexts in which these skills are expressed (e.g., see the two 4Cs evaluation grids for institutions and for games outlined in Section 4 and Section 5, below).

### 2.4. Collaboration

Collaborative problem solving—and more generally, collaboration—has gained increasing attention in national and international assessments (e.g., PISA) as an educational priority encompassing social, emotional, and cognitive skills critical to efficiency, effectiveness, and innovation in the modern global economy (Graesser et al. 2018; OECD 2017). Understanding what makes effective collaboration is of crucial importance for professional practice and training (Détienne et al. 2012; Graesser et al. 2018), as evidenced by the long line of research on group or team collaboration over the past 40 years (for a review, see e.g., Salas et al. 2004; Mathieu et al. 2017). Although there is no consensus on a definition of collaboration, scholars often see it as mutual engagement in a coordinated effort to achieve a common goal that involves the sharing of goals, resources, and representations relating to the joint activity of participants; and other important aspects relate to mutual respect, trust, responsibilities, and accountability within situational rules and norms (Détienne et al. 2012).

In the teamwork research literature, skills are commonly described across three classes most often labeled Knowledge, Behavior, and Attitudes (e.g., Cannon-Bowers et al. 1995). Knowledge competencies refer to the skills related to elaborating the knowledge content required for the group to process and successfully achieve the task/goal to which they are assigned. Behavior includes skills related to the actualization of actions, coordination, communication, and interactions within the group as well as with any other relevant interlocutors for the task at hand. Note here that effective collaboration involves skills that have also been identified elsewhere as essential competencies, including communication, creativity, and critical thinking. Finally, several attitudes have been evidenced or hypothesized as desirable competencies in the team context, for example, attitude towards teamwork, collective orientation, cohesion/team morale, etc. Another common distinction lies between teamwork and taskwork. Teamwork refers to the collaborative, communicative, or social skills required to coordinate the work within the participants in order to achieve the task, whereas taskwork refers to specific aspects related to solving the task such as using the tools and knowing the procedure, policies, and any other task-related activities (Salas et al. 2015; Graesser et al. 2018). Furthermore, collaborative competences can have specific (to a group of people or to a task) and general dimensions (i.e., easily transferable to any group or team situation and to other tasks). For example, skills related to communication, information exchange, conflict management, maintaining attention and motivation, leadership, etc. are present and transferable to a large number of group work situations and tasks (team-generic and task-contingent skills). Other skills can, on the other hand, be more specific to a team or group, such as internal organization, motivation, knowledge of the skills distributed in the team, etc.

#### 2.4.1. Individual Assessment of Collaboration

Assessing collaboration requires capturing the dynamic and multi-level nature of the collaboration process, which is not as easily quantifiable as group/team inputs and outputs (task performance, satisfaction, and changes at group/team and individual level). There are indeed multiple interactions between the context, the collaboration processes, the task processes, and their (various) outcomes (Détienne et al. 2012). The integrative concept of “quality of collaboration” (Burkhardt et al. 2009) encapsulates much of what is currently known about collaborative processes and what constitutes effective collaboration. According to this approach, collaborative processes can be grouped along several dimensions concerning communication processes such as grounding, task-related processes (e.g., exchanges of knowledge relevant for the task at hand), and organization/coordination processes (Burkhardt et al. 2009). Communication processes are most important for ensuring the construction of a common referential within a group of collaborators. Task-related processes relate to how the group resolves the task at hand by sharing and co-elaborating knowledge, by confronting their various perspectives, and by converging toward negotiated solutions. Collaboration also involves group management activities such as: (a) common goal management and coordination activities, e.g., allocation and planning of tasks; (b) meeting/interaction management activities, e.g., ordering and postponing of topics in the meeting. Finally, the ability to pursue reflexive activity, in the sense of reflecting not only on the content of a problem or solution but on one’s collaboration and problem-solving strategies, is critical for the development of the team and supports them in changing and improving their practices. Graesser et al. (2018) identify collaborative skills based on the combination of these dimensions with a step in the problem-solving process.

A large body of methodology developed to assess collaboration processes and collaborative tools has been focused on quantifying a restricted subset of fine-grained interactions (e.g., number of speakers’ turns; number of words spoken; number of interruptions; amount of grounding questions). This approach has at least two limitations. First, because these categories of analysis are often ad hoc with respect to the considered situation, they are difficult to apply in all situations and make it difficult to compare between studies. Second, quantitative variations of most of these indicators are non-univocal: any increase or decrease of them could signify either an interactive–intensive collaboration or else evidence of major difficulties in establishing and/or maintaining the collaboration (Détienne et al. 2012). Alternatively, qualitative approaches based on multidimensional views of collaboration provide a more elaborated or nuanced view of collaboration and are useful for identifying potential relationships between distinctive dimensions of collaboration and aspects of team performance, in order to identify processes that could be improved. Based on the method of Spada et al. (2005) in Computer-Supported Collaborative Learning (CSCL) research, Burkhardt et al. (2009) have proposed a multi-dimensional rating scheme for evaluating the quality of collaboration (QC) in technology-mediated design. QC distinguishes seven dimensions, grouped along five aspects, identified as central for collaboration in a problem-solving task such as design: communication (1, 2), task-oriented processes (3, 4), group-oriented processes (5), symmetry in interaction—an orthogonal dimension—(6), and individual task orientation (7). This method has recently been adapted for use in the context of assessing games as a support to collaborative skills learning.

#### 2.4.2. Institutional and Environmental Support for Development of Collaboration and Collaborative Skills

Support for individuals’ development of collaborative skills provided by institutions and programs can take a variety of forms: (a) through the social impact of the physical structure of the organization, (b) the nature of the work required within the curriculum, (c) content within the curriculum focusing on collaboration and collaborative skills, and (d) the existence and promotion of extracurricular and inter-institutional opportunities for collaboration.

For instance, institutional support for collaboration has taken a variety of forms in various fields such as healthcare, engineering, public participation, and education. Training and education programs such as Interprofessional Education or Team Sciences in the health domain (World Health Organization 2010; Hager et al. 2016; O’Carroll et al. 2021), Peer-Led Team Learning in chemistry and engineering domains (Wilson and Varma-Nelson 2016), or Collaborative Problem Solving in education (Peña-López 2017; Taddei 2009) are notable examples.

Contextual support recently arose from the deployment of online digital media and new mixed realities in the workplace, in the learning environments and in society at large—obviously stimulated and accentuated with the COVID-19 pandemic. This has led many organizations to invest in proposing support for synchronous and asynchronous collaboration (notably remote, between employees, between students and educators or within group members, etc.) in various ways, including the provision of communication hardware and software, computer-supported cooperative work and computer-supported collaborative learning platforms, training and practical guides, etc. Users can collaborate through heterogeneous hybrid collaborative interaction spaces that can be accessed through virtual or augmented reality, but also simple video conferencing or even a voice-only or text-only interface. These new spaces for collaboration are, however, often difficult to use and less satisfactory than face-to-face interactions, suggesting the need for more research on collaborative activities and on how to support them (Faidley 2018; Karl et al. 2022; Kemp and Grieve 2014; Singh et al. 2022; Waizenegger et al. 2020).

A substantive body of literature on teams, collaborative learning, and computer-supported technologies provides evidence related to individual, contextual, and technological factors impacting the collaboration quality and efficiency. For example, teacher-based skills that are critical for enhancing collaboration are, among others, the abilities to plan, monitor, support, consolidate, and reflect upon student interaction in group work (Kaendler et al. 2016). Research focuses also on investigating the most relevant tasks and evaluating the possibilities offered by technology to support, to assess (e.g., Nouri et al. 2017; Graesser et al. 2018), and/or to learn the skills involved in pursuing effective and satisfying collaboration (see e.g., Schneider et al. 2018; Doyle 2021; Ainsworth and Chounta 2021).

## 3. Labelization: Valorization of the 4Cs and Assessing Support for Their Development

Moving from the nature of the 4Cs and their individual assessment and towards the ways in which institutions can support their development in individuals, we can now address the fundamentally important question of how best to support and promote this 21st century educational mission within and among institutions themselves. This also raises the question of the systemic recognition of educational settings that are conducive to the development of the 4Cs. In response to these questions, the nature and value of labelization is now presented.

A label is “a special mark created by a trusted third party and displayed on a product intended for sale, to certify its origin, to guarantee its quality and to ensure its conformity with the standards of practices in force” (Renard 2005). A label is therefore a way of informing the public about the objective properties and qualities of a product, service, or system. The label is usually easily identifiable and can be seen as a proof that a product or service, a company, or an organization complies with defined criteria. Its effectiveness is therefore closely linked to the choice of requirements set out in its specifications, as well as to the independence and rigor of the body that verifies compliance with the criteria.

### 3.1. Labeling as a Means of Trust and Differentiation

As a sign of recognition established by a third party, the label or certification can constitute a proof of trust aiming to reassure the final consumer. According to Sutter (2005), there are different means of signaling trust. First, the brand name of a product or service and its reputation can, in itself, constitute a label when this brand name is recognized on the market. Second, various forms of self-declaration, such as internal company charters, though not statements assessed by a third party, show an internal commitment that can provide reassurance. Finally, there is certification or labeling, which is awarded by an external body and requires a third-party assessment by a qualified expert, according to criteria set out in a specific reference framework. It is this external body, a trusted third party, which guarantees the reliability of the label and constitutes a guarantee of credibility. Its objectivity and impartiality are meant to guarantee that the company, organization, product, or service meets defined quality or reliability criteria (Jahn et al. 2005).

Research on populations around the world (e.g., Amron 2018; Sasmita and Suki 2015) show that the buying decisions of consumers are heavily influenced by the trust they have in a brand. More specifically, third-party assurances and labelization have been shown to strongly influence customer buying intentions and purchasing behavior (e.g., Kimery and McCord 2002; Lee et al. 2004). Taking France as an example, research shows that quality certification is seen as “important” or “significant” by 76% of companies (Chameroy and Veran 2014), and decision makers feel more confident and are more willing to invest with the support of third-party approval than if their decision is merely based on the brand’s reputation or its demonstrated level of social responsibility (Etilé and Teyssier 2016). Indeed, French companies with corporate social responsibility labels have been shown to have higher than average growth rates, and the adoption of quality standards is linked with a 7% increase in the share of export turnover (Restout 2020).

### 3.2. Influence on Choice and Adoption of Goods and Services

Studies diverge in this area, but based on the seminal work of Parkinson (1975); Chameroy and Veran (2014), in their research on the effect of labels on willingness to pay, found that in 75% of cases, products with labels are chosen and preferred to those without labels, demonstrating the impact of the label on customer confidence—provided that it is issued by a recognized third party. Thus, brands that have good reputations tend to be preferred over cheaper new brands, because they are more accepted and valued by the individual social network (Zielke and Dobbelstein 2007).

### 3.3. Process of Labelizing Products and Services

The creation of a label may be the result of a customer or market need, a request from a private sector of activity or from the government. Creating a label involves setting up a working group including stakeholders who are experts in the field, product managers, and a certification body in order to elaborate a reference framework. This is then reviewed by a specialized committee and validated by the stakeholders. The standard includes evaluation criteria that must be clearly defined (Mourad 2017). An audit system is set up by a trusted third party. It must include the drafting of an audit report, a system for making decisions on labeling, and a system for identifying qualified assessors. The validity of the assessment process is reinforced by this double evaluation: a first level of audit carried out by a team of experts according to a clearly defined set of criteria and a second level of decision making assuring that the methodology and the result of the audit are in conformity with the defined reference framework.

### 3.4. Labelization of 21st Century Skills

The world of education is particularly concerned by the need to develop and assess 21st century skills, because it represents the first link in the chain of skills acquisition, preparing the human resources of tomorrow. One important means of simultaneously offering a reliable, independent assessment of 21st century skills and valorizing them by making them a core target within an educational system (schools, universities, and teaching and training programs of all kinds) is labelization. Two examples of labelization processes related to 21st century skills were recently developed by the International Institute for Competency Development (2021; see iicd.net; accessed on 20 November 2022) working with international experts, teachers, and researchers from the University of Paris Cité (formerly Université Sorbonne Paris Cité), Oxford University, and AFNOR UK (an accredited certification body and part of AFNOR International, a subsidiary of the AFNOR group, the only standards body in France). 

The last two or three decades has seen the simultaneous rise of international ranking systems and an interest in quality assurance and assessment in an increasingly competitive educational market (Sursock 2021). The aim of these labelization frameworks is to assist in the development of “quality culture” in education by offering individual programs, institutions, and systems additional independent, reliable means of benchmarking, charting progress, and distinguishing themselves based on their capacity to support and promote the development of crucial skills. Importantly, the external perspectives provided by such assessment system should be capable of being individually adapted and applied in a manner that can resist becoming rigidly imposed external standards (Sursock and Vettori 2017). Similarly, as we have seen in the literature review, the best approach to understanding and assessing a particular C is from a combination of different levels and perspectives in context. For example, important approaches to critical thinking have been made from educationally, philosophically, and psychologically focused vantage points (Lai 2011). We can also argue that understandings of creativity are also results of different approaches: the major models in the literature (e.g., the “4Ps” and “7Cs” models; see Lubart and Thornhill-Miller 2019) explicitly result from and include the objectives of different education-focused, process-focused, and “ingredient” or component-focused approaches.

The two assessment frameworks outlined in the sections that follow were formulated with these different perspectives and objective needs in mind. Given the complexity and very different natures of their respective targets (i.e., one assessing entire formal educational contexts such as institutions or programs, whereas the other targets the less multi-dimensional, informal educational activities represented by games), the assessment of the individual Cs also represents what experts consider a target-appropriate balance of education- and curriculum-focused, process-focused, and component-focused criteria for assessing each different C.

## 4. The International Institute for Competency Development’s 21st Century Competencies 4Cs Assessment Framework for Institutions and Programs

One comprehensive attempt to operationalize programmatic-level and institutional-level support for the development of the 4Cs is the International Institute for Competency Development’s 4Cs Assessment Framework (International Institute for Competency Development 2021). Based upon expert opinion and a review of the available literature, this evaluation grid is a practical tool that divides each of the 4Cs into three “user-friendly” but topic-covering components (see Table 1 and definitions and further discussion in the sections that follow). Each of these components is then assessed across seven dimensions (see Table 2, below), designed to cover concisely the pedagogical process and the educational context. Examples for each point level are provided within the evaluation grid in order to offer additional clarity for educational stakeholders and expert assessors.

The grid itself can be used in several important and different ways by different educational stakeholders: (1) by the institution itself in its self-evaluation and possible preparation for a certification or labelization process, (2) as an explicit list of criteria for external evaluation of the institution and its 4Cs-related programs, and (3) as a potential long-term development targeting tool for the institution or the institution in dialogue with the labelization process.

### 4.1. Evaluation Grid for Creativity

Dropping the component of “creative person” that is not relevant at the institutional level, this evaluation grid is based on Rhodes’ (1961) classic “4P” model of creativity, which remains the most concise model today (Lubart and Thornhill-Miller 2019). The three “P” components retained are: *creative process*, *creative environment*, and *creative product*. Creative process refers to the acquisition of a set of tools and techniques that students can use to enhance the creativity of their thinking and work. Creative environment (also called “Press” in earlier literature) is about how the physical and social surroundings of students can help them be more creative. Finally, creative product refers to the evaluation of actual “productions” (e.g., a piece of art, text, speech, etc.) generated through the creative process.

### 4.2. Evaluation Grid for Critical Thinking

Our evaluation grid divides critical thinking into three main components: *critical thinking about the world*, *critical thinking about oneself* (self-reflection), as well as *critical action and decision making*. The first component refers to having an evidence-based view of the exterior world, notably by identifying and evaluating sources of information and using them to question current understandings and solve problems. Self-reflection refers to thinking critically about one’s own life situation, values, and actions; it presupposes the autonomy of thought and a certain distance as well as the most objective observation possible with regard to one’s own knowledge (“meta-cognition”). The third and final component, *critical action and decision making,* is about using critical thinking skills more practically in order to make appropriate life decisions as well as to be open to different points of view. This component also addresses soft skills and attitudes such as trusting information.

Our evaluation framework for critical thinking was in part inspired by Barnett’s “curriculum for critical being” (2015), whose model distinguishes two axes: one defined by the qualitative differences in the level of criticality attained and the second comprised of three different domains of application: formal knowledge, the self, and the world. The first two components of our framework (and the seven dimensions on which they are rated) reflect and encompass these three domains. Similar to Barrett’s proposal, our third rubric moves beyond the “skills-plus-dispositions” model of competency implicit in much theorizing about critical thinking and adds the importance of “action”—not just the ability to think critically and the disposition to do so, but the central importance of training and practicing “critical doing” (Barnett 2015). Critical thinking should also be exercised collectively by involving students in collective thinking, facilitating the exchange of ideas and civic engagement (Huber and Kuncel 2016).

### 4.3. Evaluation Grid for Collaboration

The first component of collaboration skills in the IICD grid is *engagement and participation*, referring to the active engagement in group work. *Perspective taking and openness* concerns the flexibility to work with and accommodate other group members and their points of view. The final dimension—*social regulation*—is about being able to reach for a common goal, notably through compromise and negotiation, as well as being aware of the different types of roles that group members can hold (Hesse et al. 2015; Rusdin and Ali 2019; Care et al. 2016). (These last two components include elements of leadership, character, and emotional intelligence as sometimes described in other soft-skill and competency-related systems.) Participation, social regulation, and perspective taking have been identified as central social skills in collaborative problem solving (Hesse et al. 2015). Regarding social regulation in this context, recognizing and profiting from group diversity is key (Graesser et al. 2018). When describing an assessment in an educational setting of collaborative problem solving (with a task in which two or more students have to collaborate in order to solve it, each using a different set of resources), two main underpinning skills were described for the assessment: the social skill of audience awareness (“how to adapt one’s own behavior to suit the needs of the task and the partner’s requirements”, Care et al. 2016, p. 258) and the cognitive skill of planning and executing (developing a plan to reach for a goal) (Care et al. 2016). The former is included in the perspective taking and openness rubric and the latter in the social regulation component in the IICD grid. Evans (2020) identified four main collaboration skills consistently mentioned in the scientific literature that are assessed in the IICD grid: the ability to plan and make group decisions (example item from the IICD grid: teachers provide assistance to students to overcome differences and reach a common goal during group work); the ability to communicate about thinking with the group (assessed notably in the meta-reflection strand of the IICD grid); the ability to contribute resources, ideas, and efforts and support group members (included notably in the engagement and participation as well as the social regulation components); and finally, the ability to monitor, reflect, and adapt individual and group processes to benefit the group (example item from the IICD grid: students use perspective-taking tools and techniques in group activities).

### 4.4. Evaluation Grid for Communication

The evaluation grid for communication is also composed of three dimensions: *message formulation, message delivery,* and *message and communication feedback*. *Message formulation* refers to the ability to design and structure a message to be sent, such as outlining the content of an argument. *Message delivery* is about effectively transmitting verbal and non-verbal aspects of a message. Finally, *message and communication feedback* refers to the ability of students and teachers to understand their audience, analyze their social surroundings, and interpret information in context. Other components of communication skills such as theory of mind, empathy, or emotional intelligence are also relevant and included in the process of applying the grid. Thompson (2020) proposes a four-component operationalized definition of communication for its assessment in students. First, they describe a comprehension strand covering the understanding and selection of adequate information from a range of sources. Message formulation in the IICD grid captures this dimension through its focus on content analysis and generation. Second, the presentation of information and ideas is mentioned in several different modes, adjusted to the intended audience, verbally as well as non-verbally. The message delivery component of the IICD grid focuses on these points. Third, the authors note the importance of communication technology and its advanced use. The IICD grid also covers the importance of technology use in its tools and techniques category, with, for example, an item that reads: students learn to effectively use a variety of formats of communication (social media, make a video, e-mail, letter writing, creating a document). Finally, Thompson (2020) describes the recognition of cultural and other differences as an important aspect of communication. The IICD grid aims at incorporating these aspects, notably in the meta-reflection category under each of the three dimensions.

## 5. Assessing the 4Cs in Informal Educational Contexts: The Example of Games

### 5.1. The 4Cs in Informal Educational Contexts

So far, the focus has been on rather formal ways of nurturing the 4Cs. Although institutions and training programs are perhaps the most significant and necessary avenues of education, they are not the sole context in which 4Cs’ learning and improvement can manifest. One other important potential learning context is game play. Games are activities that are present and participated in throughout human society—by those of all ages, genders, and socio-economic statuses (Bateson and Martin 2013; Huizinga 1949; Malaby 2007). This informal setting can also provide favorable conditions to help improve the 4Cs (van Rosmalen et al. 2014) and should not be under-appreciated. Games provide a unique environment for learning, as they can foster a space to freely explore possibilities and one’s own potential (de Freitas 2006). We argue that games are a significant potential pathway for the improvement of the 4Cs, and as such, they merit the same attention as more formal ways of learning and developing competencies.

### 5.2. 4Cs Evaluation Framework for Games

Compared to schools and educational institutions, the focus of IICD’s evaluation framework for games (see International Institute for Competency Development 2021) is more narrow. Thus, it is fundamentally different from the institutional grid: games, complex and deep as they can sometimes be, cannot directly be compared to the complexity of a school curriculum and all the programs it contains. The evaluation of a game’s effectiveness for training/improving a given C rests on the following principle: if a game presents affordances conducive to exercising a given skill, engaged playing of that game should help improve that skill.

The game’s evaluation grid is scored based on two criteria. For example, as a part of a game’s rating as a tool for the development of creativity, we determine the game must first meet two conditions. First, whether or not the game allows the opportunity for creativity to manifest itself: if creativity cannot occur in the game, it is obviously not eligible to receive ratings for that C. Second, whether or not creativity is needed in order to perform well in the game: if the players can win or achieve success in the game without needing creativity, this also means it cannot receive a rating for that C. If both conditions are met, however, the game will be considered potentially effective to improve creativity through the practice of certain components of creative behavior. This basic principle applies for all four of the Cs.

As outlined in Table 3, below, the evaluation grid for each of the four Cs is composed of five components relevant to games that are different for each of the Cs. The grid works as follows: for each of the five components of each C, we evaluate the game on a list of sub-components using two yes/no scales: one for whether it is “possible” for that subcomponent to manifest and one for whether that sub-component is “required for success” in the game. This evaluation is done for all sub-components. After this, each general component is rated on the same two indicators. If 60% (i.e., three out of five) or more sub-components are positively rated as required, the general component is considered required. Then, the game is evaluated on its effectiveness for training and improving each of the 4Cs. If 60% or more components are positively rated as required, the game will be labelized as having the potential to be effective for training and improving the corresponding C.

The evaluation grid for creativity is based on the multivariate model of creative potential (see Section 2.1.1 and Lubart et al. 2013 for more information) and is composed of four cognitive factors and one conative factor: *originality*, *divergent thinking*, *convergent thinking*, *mental flexibility*, and *creative dispositions*. *Originality* refers to the generation of ideas that are novel or unexpected, depending on the context. *Divergent thinking* corresponds to the generation of multiple ideas or solutions. *Convergent thinking* refers to the combination of multiple ideas and the selection of the most creative idea. *Mental flexibility* entails changing perspectives on a given problem and breaking away from initial ideas. Finally, *creative dispositions* concerns multiple personality-related factors conducive to creativity, such as openness to experience or risk taking.

The evaluation grid for critical thinking echoes Halpern’s (1998) as well as Marin and Halpern’s (2011) considerations for teaching this skill, that is, taking into consideration thinking skills, metacognition, and dispositions. The five components of the critical thinking grid are: goal-adequate discernment, objective thinking, metacognition, elaborate reasoning, and uncertainty management. Goal-adequate discernment entails the formulation of inferences and the discernment of contradictions when faced with a problem. Objective thinking corresponds to the suspension of one’s own judgment and the analysis of affirmations and sources in the most objective manner possible. Metacognition, here, is about questioning and reassessing information, as well as the awareness of one’s own cognitive biases. Elaborate reasoning entails reasoning in a way that is cautious, thorough, and serious. Finally, uncertainty management refers to the dispositional propensity to tolerate ambiguity and accept doubt.

The evaluation grid for collaboration is based on the quality of collaboration (QC) method (Burkhardt et al. 2009; see Section 2.4.2 for more details) and is composed of the following five components: collaboration fluidity, well-argued deliberation and consensus-based decision, balance of contribution, organization and coordination, and cognitive syncing, input, and support. Collaboration fluidity entails the absence of speech overlap and the presence of a good flow in terms of turns to speak. Well-argued deliberation and consensus-based decision is about contributing to the discussion and task at hand, as well as participating in discussions and arguments, in order to obtain a consensus. Balance of contribution refers to having equal or equivalent contributions to organization, coordination, and decision making. Organization and coordination refers to effective management of roles, time, and “deadlines”, as well as the attribution of roles depending on participants’ skills. Finally, cognitive syncing, input, and support is about bringing ideas and resources to the group, as well as supporting and reinforcing other members of the group.

The five components used to evaluate communication in games include both linguistic, pragmatic, and social aspects. Linguistic skills per se are captured by the mastery of written and spoken language component. This component assesses language comprehension and the appropriate use of vocabulary. Pragmatic skills are captured by the verbal and non-verbal communication components and refer to the efficient use of verbal and body signals in the context of the game to achieve one’s communicative goals (Grassmann 2014; Matthews 2014). Finally, the grid also evaluates social skills with its two last components, social interactions and social cognition, which, respectively, refer to the ability to interact with others appropriately—including by complying with the rules of the game—and to the understanding of other people’ mental states (Tomasello 2005).

## 6. Discussion and Conclusions

Each of the 4Cs is a broad, multi-faceted concept that is the subject of a tremendous amount of research and discussion by a wide range of stakeholders in different disciplines, professions, and parts of the educational establishment. The development of evaluation frameworks to allow support for the 4Cs to be assessed and publicly recognized, using a label, is an important step for promoting and fostering these skills in educational contexts. As illustrated by IICD’s 4Cs Framework for educational institutions and programs, as well as its games/activities evaluation grid, the specific criteria to detect support for each C can vary depending upon the educational context (e.g., formal and institutional level or informal and at the activity level). Yet considering the 4Cs together highlights some additional observations, current challenges, and opportunities for the future that are worthy of discussion.

### 6.1. Interrelationships between the 4Cs and a New Model for Use in Pedagogy and Policy Promotion

**Figure 2:**
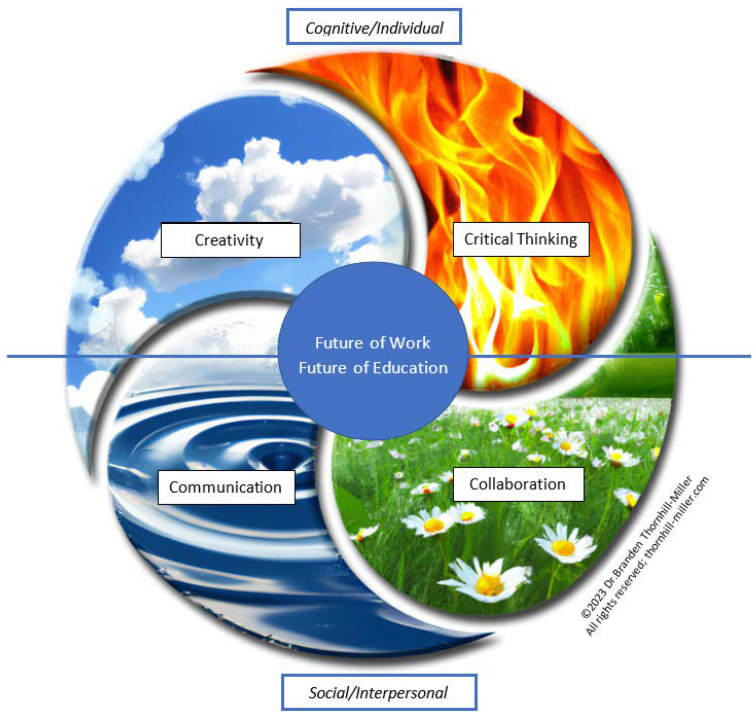
“‘Crea-Critical-Collab-ication’: a Dynamic Interactionist Model of the 4Cs”. (Illustration of the interplay and interpenetration of creativity, critical thinking, collaboration, and communication shown in dimensional space according to their differing cognitive/individual vs. social/interpersonal emphases; (© 2023, Branden Thornhill-Miller. All Rights Reserved. thornhill-miller.com; accessed on 20 January 2023)).

One very important issue for understanding the 4Cs and their educational implementation that can be simultaneously a help and a hindrance for teaching them—and also a challenge when assessing them—is their multidimensionality and interrelatedness. In other words, the 4Cs are not entirely separate entities but instead, as Figure 2 shows, should be seen as four interlinked basic “elements” for future-oriented education that can help individuals in their learning process and, together, synergistically “bootstrap” the development of their cognitive potentials. Lamri and Lubart (2021), for example, found a certain base level of creativity was a necessary but not sufficient condition for success in managerial tasks, but that high-level performance required a combination of all four Cs. Some thinkers have argued that one cannot be creative without critical thinking, which also requires creativity, for example, to come up with alternative arguments (see Paul and Elder 2006). Similarly, among many other interrelationships, there is no collaboration without communication—and even ostensibly individual creativity is a “collaboration” of sorts with the general culture and precursors in a given field. As a result, it ranges from impossible to suboptimal to teach (or teach towards) one of the 4Cs without involving one or more of the others, and this commingling also underscores the genuine need and appropriateness of assessing them together.

From this perspective, Thornhill-Miller (2021) proposed a “dynamic interactionist model of the 4Cs” and their interrelated contributions to the future of education and work. Presented in Figure 2, this model is meant to serve as a visual and conceptual aid for understanding the 4Cs and their interrelationships, thereby also promoting better use and understanding of them in pedagogical and policy settings. In addition to suggesting the portmanteau of “crea-critical thinking” as a new term to describe the overlap of much of the creative and critical thinking processes, the title of this model, “Crea-Critical-Collab-ication”, is a verbal representation of the fluid four-way interrelationship between the 4Cs visually represented in Figure 2 (a title meant to playfully repackage the 4Cs for important pedagogical and policy uses). This model goes further to suggest some dimensional differences in emphases that, roughly speaking, also often exist among the 4Cs: that is to say, the frequently greater emphasis on cognitive or individual elements at play in creativity and critical thinking in comparison to the social and interpersonal aspects more central to communication and collaboration (Thornhill-Miller 2021).

Similarly focused on the need to promote a phase change towards future-oriented education, Lucas (2019) and colleagues have suggested conflating creative thinking and critical thinking in order to propose “3Cs” (creative thinking, communication, and collaboration) as new “foundational literacies” to symmetrically add to the 3Rs (Reading, wRiting, and aRithmetic) of previous educational eras. Although we applaud these efforts, from our applied research perspective, we believe that the individual importance of, and distinct differences between, creative thinking and critical thinking support preserving them both as separate constructs in order to encourage the greatest development of each of them. Moreover, if only three categories were somehow required or preferable, one could argue that uniting communication and collaboration (as “collab-ication” suggests) might be preferable—particularly also given the fact that substantial aspects of communication are already covered within the 3Rs. In any case, we look forward to more such innovations and collaborations in this vibrant and important area of work at the crossroads between research, pedagogy, and policy development.

### 6.2. Limitations and Future Work

The rich literature in each of the 4Cs domains shows the positive effects of integrating these dimensions into educational and professional curricula. At the same time, the complexity of their definitions makes them difficult to assess, both in terms of reliability (assessment must not vary from one measurement to another) and of validity (tests must measure that which they are intended to measure). However, applied research in this area is becoming increasingly rigorous, with a growing capacity to provide the necessary tools for evidence-based practice. The development of these practices should involve interdisciplinary teams of teachers and other educational practitioners who are equipped and trained accordingly. Similarly, on the research side, further exploration and clarification of subcomponents of the 4Cs and other related skills will be important. Recent efforts to clarify the conceptual overlap and hierarchical relations of soft skills for the future of education and work, for example, have been helpful and promising (e.g., Joie-La Marle et al. 2022; Lamri et al. 2022). But the most definitive sort of taxonomy and measurement model that we are currently lacking might only be established based on the large-scale administration of a comprehensive battery of skill-measuring psychometric tests on appropriate cross sections of society.

The rapid development and integration of new technologies will also aid and change the contexts, resources, and implementation of the 4Cs. For example, the recent developments make it clear that the 4Cs will be enhanced and changed by interaction with artificially intelligence, even as 4Cs-related skills will probably, for the same reason, increasingly constitute the core of available human work in the future (see, e.g., Ross 2018). Similarly, research on virtual reality and creativity suggest that VR environments assist and expand individual and collaborative creativity (Bourgeois-Bougrine et al. 2022). Because VR technologies offer the possibility of enhanced and materially enriched communication, collaboration, and information availability, they not only allow for the enhancement of creativity techniques but also for similar expansions and improvements on almost all forms of human activity (see Thornhill-Miller and Dupont 2016)—including the other three Cs.

### 6.3. Conclusion: Labelization of the 4Cs and the Future of Education and Work

Traditional educational approaches cannot meet the educational needs of our emergent societies if they do not teach, promote, and assess in line with the new learner characteristics and contexts of the 21st century (Sahin 2009). The sort of future-oriented change and development required by this shift in institutional practices, programming, and structure will likely meet with significant resistance from comfortably entrenched (and often outdated) segments of traditional educational and training establishments. Additional external evaluation and monitoring is rarely welcome by workers in any context. We believe, however, that top-down processes from the innovative and competition-conscious administrative levels will be met by bottom-up demands from students and education consumers to support these institutional changes. And we contend that efforts such as labelizing 4C processes will serve to push educators and institutions towards more relevant offerings, oriented towards the future of work and helping build a more successful future for all.

In the end, the 4Cs framework seems to be a manageable, focused model for modernizing education, and one worthy of its growing prevalence in the educational and research marketplace for a number of reasons. These reasons include the complexity and cumbersome nature of larger alternative systems and the 4Cs’ persuasive presence at the core of a number of early and industry-driven frameworks. In addition, the 4Cs have benefitted from their subsequent promotion by organizations such as the OECD and the World Economic Forum, as well as some more direct support from recent empirical research. The promotion, teaching, and assessment of the 4Cs will require a complex social intervention and mobilization of educational resources—a major shift in pedagogy and institutional structures. Yet the same evolving digital technologies that have largely caused the need for these massive, rapid changes can also assist in the implementation of solutions (van Laar et al. 2017). To the extent that future research also converges on such a model (that has already been found pedagogically useful and policy-friendly by so many individuals and organizations), the 4Cs framework has the potential to become a manageable core for 21st century skills and the future of education and work—one that stakeholders with various agendas can already begin building on for a better educational and economic future together.

## Figures and Tables

**Table 1:** Three different components of each C in IICD’s 21st Century Skills 4Cs Assessment Framework.

**Creativity**	Creative Process	Creative Environment	Creative Product
**Critical Thinking**	Critical thinking about the world	Critical thinking about oneself	Critical action and decision making
**Collaboration**	Engagement and participation	Perspective taking and openness	Social regulation
**Communication**	Message formulation	Message delivery	Message and communication feedback

**Table 2:** Seven dimensions evaluated for the 3 different components of each C.

**Teaching Curriculum**	Aspects of the overall educational program teaching, emphasizing, and promoting the 4Cs
**Tools and Techniques**	Availability and access to different means, materials, space, and expertise, digital technologies, mnemonic and heuristic methods, etc. to assist in the proper use and exercise of the 4Cs
**Implementation**	Actual student and program use of available resources promoting the 4Cs
**Meta-reflection**	Critical reflection and metacognition on the process being engaged in around the 4Cs
**Competence of Actors**	The formal and informal training, skills, and abilities of teachers/trainers and staff and their program of development as promoters of the 4Cs
**Outside community contact**	Use and integration of the full range of resources external to the institution available to enhance the 4Cs
User Initiative *	Availability of resources for students to create and actualize products, programs, events, etc. that require the exercise, promotion, or manifestation of the 4Cs

* Educational-level dependent and potentially less available for younger students or in some contexts.

**Table 3:** Five different components evaluated for each C by the 4Cs assessment framework for games.

**Creativity**	Originality	Divergent Thinking	Convergent Thinking	Mental Flexibility	Creative Dispositions
**Critical Thinking**	Goal-adequate judgment/ discernment	Objective thinking	Metacognition	Elaborate eeasoning	Uncertainty management
**Collaboration**	Collaboration fluency	Well-argued deliberation and consensus-based decision	Balance of contribution	Organization and coordination	Cognitive syncing, input, and support
**Communication**	Social Interactions	Social cognition	Mastery of written and spoken language	Verbal communication	Non-verbal communication

## Data Availability

Not Applicable.
